# Collagen VI in the Musculoskeletal System

**DOI:** 10.3390/ijms24065095

**Published:** 2023-03-07

**Authors:** Alberto Di Martino, Matilde Cescon, Claudio D’Agostino, Francesco Schilardi, Patrizia Sabatelli, Luciano Merlini, Cesare Faldini

**Affiliations:** 1 I Orthopedic and Traumatology Department, IRCCS Istituto Ortopedico Rizzoli, 40136 Bologna, Italy; 2 Department of Biomedical and Neuromotor Science, DIBINEM, University of Bologna, 40136 Bologna, Italy; 3 Department of Molecular Medicine, University of Padova, 35131 Padova, Italy; 4 Unit of Bologna, CNR-Institute of Molecular Genetics “Luigi Luca Cavalli-Sforza”, 40136 Bologna, Italy; 5 IRCCS Istituto Ortopedico Rizzoli, 40136 Bologna, Italy

**Keywords:** collagen type VI, *COL6A1*, *COL6A2*, *COL6A3*, *COL6A4*, *COL6A5*, *COL6A6*, Ullrich congenital muscular dystrophy, Bethlem myopathy, myosclerosis myopathy, animal models, orthopedic surgery, limb deformities

## Abstract

Collagen VI exerts several functions in the tissues in which it is expressed, including mechanical roles, cytoprotective functions with the inhibition of apoptosis and oxidative damage, and the promotion of tumor growth and progression by the regulation of cell differentiation and autophagic mechanisms. Mutations in the genes encoding collagen VI main chains, *COL6A1, COL6A2* and *COL6A3*, are responsible for a spectrum of congenital muscular disorders, namely Ullrich congenital muscular dystrophy (UCMD), Bethlem myopathy (BM) and myosclerosis myopathy (MM), which show a variable combination of muscle wasting and weakness, joint contractures, distal laxity, and respiratory compromise. No effective therapeutic strategy is available so far for these diseases; moreover, the effects of collagen VI mutations on other tissues is poorly investigated. The aim of this review is to outline the role of collagen VI in the musculoskeletal system and to give an update about the tissue-specific functions revealed by studies on animal models and from patients’ derived samples in order to fill the knowledge gap between scientists and the clinicians who daily manage patients affected by collagen VI-related myopathies.

## 1. Introduction

The musculoskeletal system has the primary functions of supporting the body, allowing motion, and protecting vital organs [1]. It is composed of the skeleton’s bones, cartilage, tendons, ligaments, and other connective tissue that supports and connects the different counterparts constituting the joints. Motion around the joints is powered by skeletal muscles, connected to neighboring bony structures, able to transduce the force generated by muscle contraction. Through contraction, muscles allow the motion of bones attached to them by tendons. While bones provide support to the body, joints ensure the connections of contiguous bones; the cartilage at that level prevents the bone ends from rubbing directly onto each other, while ligaments connect bones and contribute to articular stabilization [1].

The word “connective tissue” refers to the tissue that supports and ties tissues and organs. It consists of fibroblasts and their surrounding extracellular matrix (ECM). The ECM is crucial for cell adhesion, stability, and regeneration in different tissues [1]. An important role is played by the different types of collagens allowing the formation of a structure that supports a remarkably wide range of tissues. Among the different types of collagens typically involved in the formation of the ECM (including types I, II, III, VI, IX, and XI), collagen VI (colVI) is expressed as a ubiquitous ECM protein in the stroma, forming a microfibrillar network associated with the basement membrane [2].

ColVI exerts several functions in the tissues in which it is expressed, ranging from mechanical roles, which are typical of collagen components of the ECM, to more specific cytoprotective functions, including the inhibition of apoptosis and oxidative damage, the promotion of tumor growth and progression, and the regulation of cell differentiation and autophagic mechanisms; moreover, it is involved in the maintenance of cell stemness [3]. ColVI monomer is made by the assembly of three different chains, α1(VI), α2(VI), and α3(VI), the latter alternatively substituted by α5(VI) or α6(VI) in humans, in a tissue-specific manner [4,5]. Mutations in the genes encoding its main chains, *COL6A1*, *COL6A2* and *COL6A3*, are responsible for the spectrum of congenital muscular disorders, namely Ullrich congenital muscular dystrophy (UCMD), Bethlem myopathy (BM), and myosclerosis myopathy (MM), showing a variable combination of muscle wasting and weakness, joint contractures, distal laxity, and respiratory compromise [6,7,8]. These diseases differ in terms of clinical appearance and severity of the disorder. UCMD has a more severe pattern, and it is characterized by early-onset weakness and proximal contractures, as well as by pronounced distal joint hyperlaxity. Patients in most severe cases do not achieve ambulation, or it can be achieved only for a limited period of time [9]. BM has milder manifestations; patients usually ambulate well in adulthood, albeit the typical joint contractures are prominent [9]. MM is characterized by early-onset widespread and progressive muscle and joint contractures, paralleled by “woody” and atrophic muscle consistency, causing a severe limitation of motion [8]. Despite the depth of our knowledge about these diseases, no effective therapeutic strategy has been developed so far [10]; moreover, the effects of colVI mutations on other tissues have been poorly investigated so far.

The aim of this review is to outline the role of colVI in the musculoskeletal system and to give an update about the tissue-specific functions revealed by studies on animal models and from samples of patients affected by the above-mentioned diseases. The main objective is to fill the knowledge gap between scientists and the clinicians who daily manage patients affected by collagen VI-related myopathies, as well as to provide an easy consultation guide and a source to plan and drive further research on this topic.

## 2. Collagen VI Expression, Assembly, and Secretion

ColVI formation requires the harmonic expression of different genes. Six independent genes were found conserved between mice and humans that are responsible for the expression of colVI α-chains. By mapping the human genome, the genes have been located on three chromosomes: the *COL6A1* and *COL6A2* genes coding for α1(VI) and α2(VI), respectively, are located on chromosome 21q22.3 [9]; on chromosome 2q37 lies the *COL6A3* gene; and three other alternative chains of α3(VI) are transcribed by the *COL6A4*, *COL6A5*, and *COL6A6* genes that are mapped on chromosome 3q22.1 [4]. The human *COL6A4* gene, due to chromosome inversion, transcribes for noncoding pseudogenes [4].

In the N- and C-terminal globular regions of all colVI chains, a von Willebrand factor type A (vWF-A) module flanks the triple-helix domain of 335–336 amino acid residues. The α1(VI) and α2(VI) chains are 130–150 kDa in size, with one N-terminal and two C-terminal vWF-A modules (N1, and C1 and C2, respectively) [11]. The α3(VI), α5(VI) and α6(VI) chains are considerably larger, ranging from approximately 220 kDa to over 300 kDa. Despite structural similarities at the N-terminal end, these show two C-terminal vWF-A modules (C1 and C2) followed by distinctive regions (C3, C4, and C5). The C3 domain includes a proline-rich sequence; C4 is a fibronectin-type-III domain; and C5 is a Kunitz-like domain [12].

Several alternatively spliced variants that involve some of the vWF-A modules and the N-domains have been described in mouse tissues and human cells [13,14]. Most frequently in humans, the alternative splicing of α2(VI) can produce three different protein variants, known as α2C2, α2C2a and α2C2a′ [15].

Early studies described colVI as being constituted by the α1(VI), α2(VI), and α3(VI) chains, which assemble into a triple-helix monomer (≈500 kDa) [16,17,18,19,20,21]. The short triple-helix formation is favored by the presence of Gly-X-Y triplets; the presence of proline in the Y position, potentially modified to hydroxyproline [22], is able to confer most of the stability to the triple helix [23]. A 1:1:1 stochiometric ratio for the α1(VI), α2(VI), and α3(VI) chains in forming a heterotrimeric monomer is strongly supported by in vitro studies performed by Lamandé et al. [24] in a particular cell line expressing high levels of mRNA encoding for α1(VI) and α2(VI), but not α3(VI). As a result, the cell line did not produce a colVI triple helix, demonstrating that α3(VI) expression was essential for the assembly of a colVI monomer [24]. Recently, Gara et al. [4] identified three novel chains, α4(VI), α5(VI), and α6(VI), that share a similar structure to the α3(VI). These polypeptides allow the variable assembly of colVI triple-helix monomers as α1–α2–αX (where αX could alternatively be α3, α4, α5, or α6) [4].

Prior to secretion, colVI goes through a rigorous multistep intracellular assembly process into larger aggregates [25]. The intracellular assembly involves the formation of antiparallel dimers (≈1000 kDa), which assemble into tetramers (≈2000 kDa), both stabilized by disulfide bonds [26]. Finally, these tetramers are released into the extracellular space, where they link end-to-end via non-covalent interactions to create the peculiar 105 nm beaded microfilaments visible by electron microscopy [21,27].

Globular domains of colVI not only contribute to monomer assembly [28] and further to dimer [25] and tetramer [28] association and stabilization but are also pivotal in granting heterotypic interactions with several ECM molecules in the extracellular environment [29,30,31], although triple helical regions also mediate some of these interactions [32,33]. The major colVI interactors among ECM proteins include hyaluronan and heparin [34], fibrillar collagens such as collagen I [12], collagen II [31], basement membrane-associated collagen IV [29], fibronectin [35] and the proteoglycans biglycan, decorin [36], and perlecan [32] (a wider description is provided in Cescon et al., 2015 [3]). Nonetheless, the colVI superstructure is supposed to favor the engagement of specific membrane protein receptors. While some of these were experimentally identified, the search for the colVI receptors transducing its signal intracellularly is still an open field of research.

## 3. Collagen VI in Tissues Relevant for the Musculoskeletal System

The ubiquitous expression of colVI and its interaction with ECM constituents suggest that it plays a key role in the repair, development, and homeostasis processes in a multitude of tissues [37,38].

**Figure 1:**
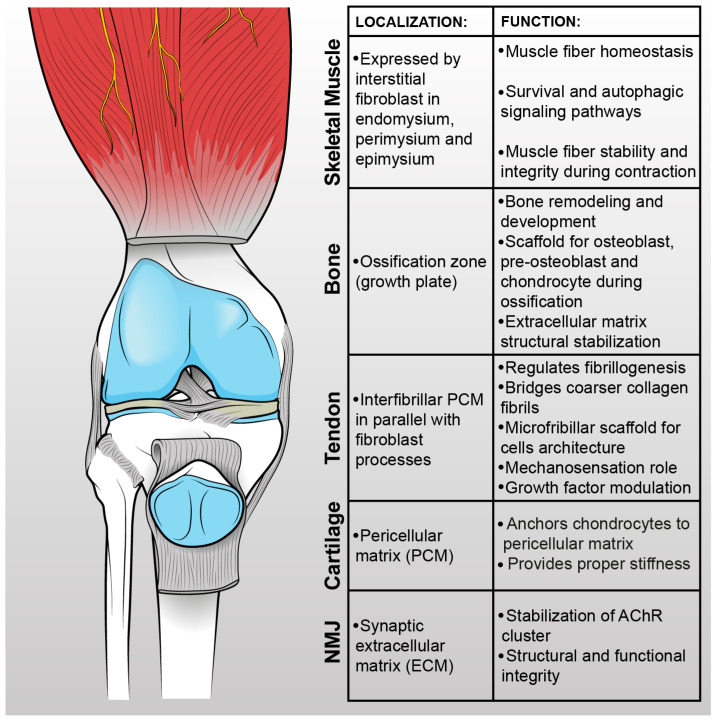
The role of collagen VI in different tissues: *Skeletal Muscle*, *Bone*, *Tendon*, *Cartilage and Neuromuscular Junction (NMJ)*.

**Figure 2:**
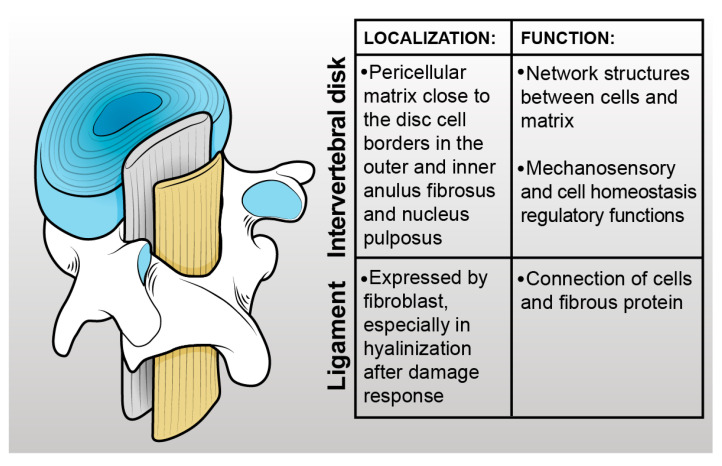
The role of collagen VI in different tissues: *Intervertebral disk and Ligament*.

With relevance for the musculoskeletal system, current knowledge about the role of colVI in skeletal muscle, bone, tendon, cartilage, and ligament tissue will be presented in subsequent sections. A schematic description is also provided in Figure 1 and Figure 2.

## 4. Skeletal Muscle

### 4.1. General Aspects

Muscle tissue is a highly specialized contractile tissue found in the human body in three types: smooth, cardiac, and skeletal [1].

Skeletal muscle is composed of muscle cells and myoblasts that, during development, fuse into a multinucleated muscle fiber. Thanks to the arrangement of sarcomeres, this muscle fiber can contract and shorten along a longitudinal axis. Muscle fibers are surrounded by a layer of connective tissue, called the endomysium. Muscle fibers associated in muscle bundles are enclosed by a further layer of connective tissue, the perimysium, while a third layer, called the epimysium, enwraps the whole muscle structure and is in continuity with the deep fascia, separating muscles from other surrounding tissues. These layers of connective tissues, beyond their containment role, are pivotal elements in the transmission of skeletal muscle force produced by myofiber contraction [39].

ColVI is a major component of the skeletal muscle ECM; it is diffusely expressed in the different layers of muscle connective tissue, including the endomysium, perimysium, epimysium, and deep fascia [40]. As visualized by the electron microscope immunogold technique, colVI microfilaments distribute close to the collagen and fibronectin fibrils [41] of the muscle interstitium and form a dense network in the reticular lamina of the muscle fibers, connecting the basal lamina with the fibrillar collagen of the ECM [30,41]. Interestingly, by using single-chain-specific antibodies, the α3 chain appears ubiquitous, the α6 chain is less expressed and mainly interstitial, while the α5 chain is selectively expressed at the myotendinous junctions. The restricted and differential distribution pattern of the α5 and α6 chains, with respect to their homologue α3 chain, implies that different colVI isoforms may have specific roles in specialized muscle ECM structures. A unique critical role of α3 chain-based heterotrimers is at the level of the muscle fiber basement membrane [42], where, by interacting with ECM components and sarcolemmal receptors, they may contribute to muscle fiber stability and integrity during contraction.

Interstitial fibroblasts are the major cell type responsible for the deposition of colVI [43], whereas myogenic cells, which produce factors that can influence collagen secretion, do not express it [44], with the exception of quiescent satellite cells that express colVI until they become activated [45]. Interestingly, colVI was recently described as being part of the highly specialized ECM within the synaptic cleft of the neuromuscular junction (NMJ) [46].

The crucial role of colVI in skeletal muscle is emphasized by the fact that mutations in the genes encoding its chains have a causative role in several forms of inherited human muscle diseases, including BM, UCMD, and MM [8,47,48] as previously described.

### 4.2. Animal Models

Initial studies aiming at understanding the role of colVI in vivo led to the production of the first murine model with inactivation of the *Col6a1* gene, the *Col6a1*^−/−^ mouse, in which the ablation of the major α1(VI) chain results in the complete absence of colVI secretion in all the tissues studied so far [49]. Analyses performed on the *Col6a1* null mice revealed a cytoprotective role of colVI since the ablation of the protein triggered spontaneous apoptosis in muscle fibers that were associated with latent mitochondrial dysfunction and organelle alterations [50]. In the colVI knockout muscle, an increased opening of the permeability transition pore (PTP) in the mitochondrial inner membrane was highlighted as causative for the dystrophic phenotype [27,51] and became a major successfully targeted downstream defect by the therapeutical approaches tested thus far [51,52]. Mitochondrial alterations were paralleled by increased oxidative damage and reactive oxygen species (ROS) production [53]. Therapeutic approaches were successfully designed, targeting the increased level of ROS activity found in colVI null muscles and depending on the raised activity of mono-amino-oxidase (MAO), an enzyme located on the external mitochondrial membrane and responsible for myofibrillar protein oxidation. MAO inhibition, by its inhibitor pargyline, was tested in mice and was able to ameliorate the muscle phenotype [53].

Subsequent studies demonstrated that the occurrence of altered mitochondria in *Col6a1*^−/−^ muscle is due to defects in the regulation of the autophagic pathway and that autophagy flux reactivation by pharmacological, genetic, or nutritional approaches is able to rescue the occurrence of dysfunctional organelles, apoptosis, and muscle strength [54,55,56]. Interestingly, the same features, namely motility impairment, altered organelle ultrastructure, and defective autophagy were also described in a novel mutant colVI knockout zebrafish, throwing light on the role of colVI during embryonic development and rendering the mentioned features as candidate targets in high-throughput drug screening approaches [57].

ColVI was found to have a role in the niche of muscle stem cells. The colVI genes are not only expressed by isolated quiescent (but not activated) satellite cells but are also overexpressed in skeletal muscle upon injury, triggered by cardiotoxin. In this condition, Urciuolo and colleagues demonstrated that *Col6a1*^−/−^ mice had impaired satellite cell self-renewal capabilities and defective muscle regeneration [45]. In this context, a role for colVI in regulating muscle stiffness was unraveled by demonstrating a lower stiffness in *Col6a1*^−/−^ muscles at a level that both in vivo as well as in vitro, when recreated by engineered structures, promotes satellite cell differentiation rather than sustaining self-renewal [45].

The in vivo function of colVI at the NMJ was also investigated in adult *Col6a1*^−/−^ mice. Fluorophore-coupled α-bungarotoxin (αBT), which specifically binds acetylcholine receptors (AChRs), allowed monitoring of the morphology of post-synaptic boutons on different muscles, showing greater fragmentation than wild-type, a typical hallmark of denervation or aging and altered expression level for a panel of NMJ-enriched proteins at the post-synaptic apparatus [46]. This was paralleled by an increase in the expression of the fetal *Chrng* and activity-dependent *Chrna* genes, but not *Chrne*, indicating NMJ changes and an abnormal neuromuscular transmission, further demonstrated by electrophysiological analysis [46]. Of note, studies performed on colVI knockout zebrafish demonstrated a reduced motor axon elongation and branching within the myotome at both 24 and 48 h of development, thus revealing an early defect in muscle innervation and, in turn, impairing mutant fish motility [57].

Despite the severe effects of colVI ablation on muscle fibers and cell-survival pathways like apoptosis and autophagy, the specific cell receptors that mediate these effects in muscle from colVI are still unclear [50,54]. Early in vitro studies identified several cell surface receptors that are capable of binding colVI, including α1β1, α2β1, α3β1, α10β1, and αvβ3 integrins [58,59,60], as well as the chondroitin sulfate proteoglycan-4 (CSPG4; also known as NG2) [61,62,63] and, more recently, the anthrax toxin receptor 2 (Antxr2/CMG2) [64]. However, how colVI extracellular signals are transduced within muscle fibers remains unknown.

### 4.3. Clinical Studies

In patients affected by BM, symptoms differ from the onset of the disease. Patients with prenatal-onset BM usually show decreased fetal movements, congenital torticollis or bilateral clubfoot. Hypotonia and delayed motor milestones are the clinical typical presentation of neonatal-onset BM. More often, patients with early-childhood-onset BM present limb-girdle weakness or joint contractures. Sometimes in adult-onset BM, contractures can define the clinical presentation and overshadow muscle weakness [65]. Patients present systemic muscular atrophy and weakness affecting proximal muscles more than distal ones and extensors more than flexors. Contractures of the interphalangeal joints of the last four fingers as well as flexion contractures of the elbow and ankles are the most common clinical symptoms [66].

Otherwise, patients affected by UCMD manifest proximal joint contractures, striking distal hyperextensibility, and protruding calcanei [67,68]. Contractures of proximal joints, congenital torticollis, and delayed motor developmental milestones are other common clinical presentations [69]. Muscular weakening and atrophy are systemic and gradually progressive. Hyperextensibility of distal appendicular joints and flexor muscle weakness are common clinical signs [70].

Alterations in skeletal muscles underlie the typical clinical symptoms of patients affected by BM and UCMD. Different research groups [27,50,51,52], through the study of muscle biopsies and myoblast cultures, assessed the role of a Ca^2+^-mediated mitochondrial dysfunction in the pathogenesis of muscle fiber death. Functional and ultrastructural mitochondrial abnormalities are due to improper opening of the permeability transition pore, a mitochondrial inner membrane channel [52]. Moreover, the inadequate removal of defective mitochondria and the increased apoptosis amplify the damage [27].

This evidence laid the foundation for the latest clinical trials, and aiming at the downstream targets of genetic defects is still a major focus in colVI-RM therapeutic approaches.

In one of the latest clinical trials, Merlini and colleagues [10,71] demonstrated how these typical mitochondrial alterations could be controlled using Cyclosporin A, an immunosuppressant that desensitizes the permeability transition pore independently of calcineurin inhibition [72]. In accordance with studies in mice, patients’ myoblasts were also found to be more sensitive to oxidative damage in cultures, and the inhibition of MAO activity by pargyline significantly reduced both ROS accumulation and mitochondrial dysfunction [73].

Moreover, colVI deficiency in mice results in defective autophagy flux regulation and sustained activation of the Akt–mTOR pathway under fasting conditions, as well as reduced levels of Beclin 1 and BNIP3, which are essential effectors in autophagy initiation. Indeed, muscle biopsies from UCMD and Bethlem myopathy patients exhibited a significant drop in Beclin 1 and BNIP3 protein levels, demonstrating a failure in autophagy regulation in collagen VI-related myopathies [54]. Such an aspect was also approached in a clinical trial aiming at testing a low-protein diet for the reactivation of autophagy in BM/UCMD patients, leading to increased autophagic markers and reduced apoptosis in patients’ biopsies, with evidence of benefits for muscles and metabolic modulation towards an improved mitochondrial homeostasis, as well as to the identification of blood leukocytes as a worthy noninvasive tool to monitor autophagy in patients [74].

Clinical observations also highlighted the relevance for the role of colVI in satellite cells and at the NMJ. Indeed, a defect in the regenerative potential of satellite cells upon the lack of colVI is consistent with our study [75] and other works [76] on UCMD muscle biopsies, which reported prominent but abnormal regeneration in UCMD muscle biopsies. Moreover, muscle biopsies isolated from patients with different colVI gene mutations and clinical phenotypes ranging from typical BM to severe UCMD, showed significantly greater colVI and utrophin expression at the level of NMJs than in unaffected control biopsies. This finding is consistent with the *Col6a1^−/−^* mice abnormality. Moreover, UCMD biopsies revealed elevated mRNA levels of the CHRNG and CHRNA genes (which code for the acetylcholine receptor subunits) when compared with unaffected control biopsies, similarly to *Col6a1^−/−^* mice [46].

## 5. Bone

### 5.1. General Aspects

ColVI is an important bone matrix protein [77] that is linked to bone remodeling and bone development [78,79]. Its deficiency alters the configuration and the shape of osteoblasts in both the inner periosteal layer and the cancellous bone, which affects the maintenance of bone mass because osteoblasts require a regular morphology to work properly [77]. Bone forms the skeleton—the structure that gives shape to the body, supports its weight, and facilitates locomotion. Bone is also able to react elastically to mechanical forces. If it breaks, and under the appropriate conditions, it heals without leaving scars, regaining its original shape [1]. Bone is a highly dynamic tissue whose formation and structural integrity are regulated by specialized cell types: osteoblasts regulate the anabolic processes that lead to new bone formation and closely collaborate with osteoclasts, which have the catabolic role of resorbing bones. This role-play is at the basis of the bone turnover, which is the process that replaces and renews micro-damaged bone tissue from impact or aging [80]. An imbalance in bone turnover can lead to osteopetrosis (an excess of bone matrix) or osteopenia (insufficient bone matrix). In both cases, the bone is fragile and at risk of impending fractures [81,82]. The most prevalent type of collagen in bone is type I collagen, which is made up of two α1(I) and one α2(I) chains joined together to form a triple-helix structure. The clinical manifestations of individuals with mutations in type I collagen chains or in the enzymes that control collagen maturation demonstrate the significance of type I collagen in bone, as in the case of brittle bone disease, also known as osteogenesis imperfecta [83]. Collagen I is an important regulator of osteoblast differentiation and a guide for the calcification of bone tissue [84]. In this scenario, colVI appears to be involved in ECM structural stabilization via direct interactions with other macromolecules, including collagen I. In the initial stages of IL-4-induced mineralization, colVI plays a role in controlling collagen I expression [85]. In normal human periosteal osteoblast-like cells (named SaM-1 cells), interleukin-4 increased collagen I and osteocalcin accumulation and caused mineralization [86]. ColVI was reported to be expressed by SaM-1 cells. The microenvironment of osteoblasts and their surrounding matrix would be mediated by colVI, which is produced by osteoblasts [77]. The rhIL-4 used in the studies [87,88] increased the expression of α1(VI) and α2(VI) collagen mRNA and the deposition of colVI in the cell layer, suggesting that an accumulation of colVI in the surrounding ECM may modulate the upregulation of collagen I mRNA in rhIL-4 treated SaM-1 cells [87,88]. In the early stages of osteoblastic differentiation, colVI may act as a matrix-derived inducer of integrin-mediated collagen I expression [85].

### 5.2. Animal Models

Several researchers investigated the hypothesis that the absence of colVI affects bone formation and age-related alterations.

After an initial observation that colVI deficiency led to delayed ossification in one-month-old mice and general decreased bone mineral density in adults [89], Izu et al. [77] studied how osteoblasts and bone structure react upon colVI depletion in four-week-old *Col6a1^−/−^* and wild-type mice. According to 3D-μCT images, the secondary trabecular bone in the metaphyseal area of the femur showed a sparse pattern due to the colVI deficit. The levels of bone volume in cancellous bone decreased by approximately 30% in *Col6a1^−/−^* mice [77]. Histologically, the effects of colVI deficit on the morphology of osteoblasts were investigated. In cancellous bone from wild-type mice, osteoblastic cells were seen with a cuboidal form in a single layer on the surface of the trabecular bone at hematoxylin and eosin staining. These cells also showed an ovoid nucleus filled with cytoplasm, which is a common characteristic of osteoblastic cells secreting bone matrix. Conversely, in *Col6a1^−/−^* mice, osteoblasts on the surface of the trabecular bone displayed irregular cellular borders, and the shape of the osteoblasts in the cortical bone was flattened. Moreover, osteoblasts appeared chaotically organized, and the border between these and the bone matrix was severely disrupted, which shifted from having a straight look in wild-type to a wavy appearance in knockout mice [77]. The study also looked at the bone’s dynamic metabolic characteristics. Based on the results of the investigation utilizing calcein double labeling, bone formation parameters were investigated. Although not statistically significant, the mean values of the rates of mineral apposition and bone formation were lower in *Col6a1^−/−^* than in wild-type mice [77].

The knee joints of the *Col6a1^−/−^* mice were another subject of the earliest in vivo investigation conducted by Christensen et al. [78] that looked at the role of colVI in bone structure and physical characteristics. By 3D-μCT performed in the trabecular regions of the tibial epiphysis, they found that, compared with wild-type mice whose trabecular bone volume increased noticeably with age, the trabecular bone volume of *Col6a1^−/−^* mice remained smaller and steady. Connectivity density decreased noticeably as *Col6a1^−/−^* mice aged, while it remained consistently greater in wild-type mice. Bone tissue density increased only 4% with skeletal maturity from 2 to 9 months in *Col6a1^−/−^* mice, whereas in wild-type mice bone tissue density increased 19% [78]. Bone volume and bone tissue density were measured by 3D-μCT at the tibial metaphysis. Both displayed a statistically significant main effect of age (but not genotype) and a statistically significant age-by-genotype interaction effect. In contrast to wild-type mice, whose bone volume had an increase of 77% from 2 to 9 months, the bone volume of *Col6a1^−/−^* mice volume did not alter appreciably overtime. Metaphyseal tissue density in wild-type mice had an increase of 34% compared with 7% in *Col6a1^−/−^* mice [78]. Finally, *Col6a1^−/−^* mice did not gain any mineral content with age [78]. All these findings clearly indicated that substantial skeletal disorders are linked to colVI deficiency.

It is well established that mechanical loading is critical for bone development and maturation [78]. For these reasons, due to the myopathy of *Col6a1^−/−^* mice, inappropriate knee loading may exist and may affect the properties of trabecular bone [49]. The variations between *Col6a1^−/−^* and wild-type trabecular bone formations closely reflect the pattern of alterations discovered in the research on limited locomotor modes [78], mechanically-altered loading [90], or disuse [91].

More recently, Pham and colleagues [83] used 3D-μCT to measure femoral and vertebral bone mass in a different model, the *Col6a2*^−/−^ mouse. ColVI expression in bone was evaluated using immunofluorescence labeling in wild-type and *Col6a2^−/−^* mouse bones and was found to be strong at the level of the hypertrophic cartilage layer next to newly produced bone in the primary ossification center in one-month-old wild-type mice, while absent in the knockout ones. *Col6a2^−/−^* mice were marginally smaller than their age- and sex-matched wild-type counterparts, with DEXA measurement revealing a reduced whole-body bone mineral density (BMD) compared with wild-type [83], in agreement with findings previously published by Alexopulos and colleagues in the *Col6a1^−/−^* mice [89]. To determine how *Col6a2^−/−^* mouse bones were affected by colVI deficiency, isolated femora and vertebrae (L3) were dissected and µCT analyzed. Both the femur and the L3 vertebrae of three-month-old *Col6a2^−/−^* mice showed significantly reduced BMD, reduced bone volume upon the whole tissue volume (BV/TV), smaller numbers of trabeculae, and higher trabecular spacing compared with wild-type mice of the same age [83]. These variations persisted with age and were still visible in six-month-old mice [83]. In mice lacking *Col6a2*, cortical bone was not affected. When the middle diaphyseal cortical femur was examined for modifications, micro-CT examinations revealed no glaring differences in the cortical morphology. Quantification of the cortical area revealed no appreciable differences in the diameter of the diaphysis, in medullary diameter, in BV/TV, cortical thickness, cortical porosity, or BMD between the *Col6a2^−/−^* and wild-type controls [83].

The skeletal phenotype of mice deficient in α2(VI) protein was further analyzed, demonstrating how colVI governs the balance between bone formation and resorption to regulate trabecular bone mass in both femur and spine. *Col6a2^−/−^* mice exhibit enhanced osteoclastogenesis but no change in bone formation characteristics. Dynamic histomorphometry using a double fluorochrome labeling technique was used to identify the cellular basis for the reduced bone mass phenotype observed in *Col6a2^−/−^* mice. There were no significant differences in mineral apposition rate, mineralizing perimeter, or bone formation rate between *Col6a2^−/−^* and wild-type mice [83]. The osteoblast-expressed genes osteopontin (*Spp1*) and osteocalcin (*Bglap*) had identical relative mRNA expression levels in wild-type and *Col6a2^−/−^*-derived cells. Using fluorescence-based staining to examine osteoclastogenesis levels in bones, *Col6a2^−/−^* animals exhibited significantly more positive staining than wild-type mice in the number of osteoclasts per trabecular length. The expression of an osteoclast-associated Ig-like receptor and Cathepsin K revealed that the relative levels of osteoclast-expressed genes were substantially higher in the *Col6a2^−/−^* bones [83].

The dysregulation of bone remodeling pathways was revealed by RNA sequencing analysis that was performed on bones from wild-type and *Col6a2^−/−^* mice, supporting an enhanced osteoclastogenesis and a potential connection to TNF-α.

The quantity of myeloid osteoclast precursors was not affected in *Col6a2^−/−^* mice, but the response to TNF-α was increased in osteoclast precursors of *Col6a2^−/−^* mice. TNF-α was found to be an upstream regulator in *Col6a2^−/−^* cells by further analysis of the RNA sequencing data. Pham and colleagues [83] treated wild-type osteoclast progenitors with or without conditioned media from wild-type or *Col6a2^−/−^* BMSCs (bone marrow stroma cells) to see if TNF-α activity was altered. TNF-α response was more effective in BMSCs treated with conditioned medium from *Col6a2^−/−^* cells than in the other sets, indicating that TNF-α plays a role in the hyperactive osteoclastogenesis reported in *Col6a2^−/−^* mice [83].

In vitro, α2(VI) directly bound to TNF-*α* and inhibited its ability to cause osteoclastogenesis in osteoclast precursors exposed to TNF-*α*: a dose-dependent response was observed in recombinant human COL6A2 fragment binding when the experiment was conducted on TNF-*α*-coated plates. If an osteoclast precursor cell-line was treated with either RANKL or TNF-α to enhance osteoclastogenesis or with colVI, it was demonstrated that TNF-*α* increased RANKL-induced osteoclastogenesis and colVI decreased osteoclast quantity and differentiation in RANKL and TNF-*α*-induced cells. ColVI binds to TNF-α and inhibits its capacity to promote osteoclastogenesis. Therefore, when *Col6a2* was depleted, TNF-*α* was not trapped in the ECM and was free to boost RANKL’s effects on osteoclastogenesis [83].

### 5.3. Clinical Studies

In the literature to date, there are few clinical studies focusing on bone in patients affected by colVI-RM. However, osteopenia is a commonly reported finding in patients with BM or UCMD, as well as bone deformities (e.g., high-arched palate, congenital hip dislocation, protruding calcanei, torticollis, and kyphotic spine deformity) [92]. As early as Ullrich’s initial description, osteopenia was found in several cases [68,93].

In the study conducted by Toni and colleagues [92], patients with BM and UCDM underwent dual-energy X-ray absorptiometry (DXA). By calculating the T-score, the analysis showed that the bone mineral content is greatly reduced in these patients, especially in men.

Finally, Philippe et al. [94] analyzed bone mineral density in a group of adult patients with muscular dystrophies and noted greatly reduced values compared with the general population, especially in wheelchair-bound patients. Although those patients were affected by different syndromes, their clinical conditions and disabilities are similar to those affected by BM and UCDM.

## 6. Tendon

### 6.1. General Aspects

Tendons are bands of fibrous connective tissue that connect a muscle to a bone, allowing the transmission of force generated at the muscle level. Each muscle is characterized by at least two tendons, one proximal and one distal. The transition area between muscle and tendon tissue is called the myotendinous junction, while the area of insertion at bone level is called the osteotendinous junction [1].

Tendons transfer mechanical stresses from muscle to bone; these are composed of fibroblast columns (tenocytes) with collagen fibrils serving as the primary structural component. The majority of fibrils are composed of collagen type I, but they also contain glycoproteins, proteoglycans, collagen types III, V, VI, XII, and XIV, as well as other substances that aid in the process of fibrillogenesis. Fibrils are arranged uniaxially into fibers; tenocytes and fibers join together to form fascicles, which are then linked together by connective tissue sheaths to form the mature, weight-bearing tendon [95,96,97].

ColVI is found in the interfibrillar tendon’s extracellular matrix (ECM), where it acts as a link among the coarser collagen fibrils. In addition, it is enriched in the tenocyte’s pericellular matrix (PCM), where it forms a microfibrillar scaffold critical for cell behavior and function [98]. Because of its cell–matrix and matrix–matrix interactions, colVI acts as a critical regulator of matrix signals [29,99]. Moreover, it influences collagen fibril construction and tendon function. ColVI may function as a “bridge” between cells and the ECM, playing important roles in cell mechanosensation and cell surface growth factor modulation [100].

Patients suffering from collagen VI-related myopathies have axial and proximal joint contractures, as well as distal joint hypermobility, indicating that tendon structure and function are compromised.

### 6.2. Animal Models

The functional roles of colVI in tendons were investigated using a mouse model with a specific deletion of the *Col6a1* gene. The flexor digitorum longus (FDL) tendons of *Col6Ia1*^−/−^ and wild-type mice were dissected at P1, P7, P16, and P30. ColVI was immuno-localized in wild-type FDL tendons on days P1, P7, P16, and P30 of development. Its reactivity was increased in the pericellular area of tendon fibroblasts, localizing within and around tendon fibroblasts at the level of the fibroblast processes, suggesting a role for colVI in tendon formation at the fibroblast–matrix interface.

The morphological characteristics of *Col6a1^−/−^* and wild-type growing tendons were studied using TEM of FDL tendons at P1 and P30. At an early stage of tendon formation, wild-type tendon fibroblasts were physiologically structured and produced well-defined fibers (P1). Tendon fibroblasts aided in the formation of micro-domains in which fibers were assembled; this arrangement was disrupted in P1 *Col6a1^−/−^* tendons, displaying altered fibroblast cell shape and organization. This resulted in disordered micro-domain organization and disruption of tendons fibers structure in *Col6a1^−/−^* tendons. Of note, micro-domain structure and fiber organization remained abnormal as tendon development progressed (P30).

Smaller and less structured fibers were present in *Col6a1^−/−^* tendons than in wild-type. Thus, it is thought, that col VI plays a key role in tendon development by maintaining cell shape and fiber organization.

At time P1, no differences were evident in the tendons of *col6a1^−/−^* and wild-types. Conversely, at P30, structural and fibrillar organization differences were shown in *Col6a1^−/−^* pericellular area [101].

Fibrillar structure and fibrillar diameter variation at the pericellular region and regions displaced from the cells to the fiber’s center were examined. Fibrils in *Col6a1^−/−^* tendons had a smaller diameter than those in wild-type tendons in both areas; in wild-type tendons, on the other hand, fibrils of larger diameter and uniform distribution were present. Thus, colVI deficiency resulted in an alteration in fibrillar assembly in the pericellular region and the following fibrillar growth, leading to less tendon thickness in *Col6a1^−/−^* mice. This lower tendon thickness obviously affects the biomechanical properties of the tendon, resulting in lower tensile strength and less stiffness of the tendon in *Col6a1^−/−^* mice compared with wild-type [101].

The study of a colVI null mouse model demonstrated faulty regulation of tendon fibrillogenesis. There was a disruption in cell and extracellular micro-domain organization in the absence of colVI [101]. The breakdown of micro-domain structure seen in *Col6a1^−/−^* tendons was linked to abnormal fiber development within these micro-domains. Finally, in the absence of colVI, there was abnormal fibril assembly in the pericellular area as well as abnormally small diameter fibril assembly throughout the tendon [101]. The abnormal structure of the tendon fibrillar matrix was linked to altered function, lower tensile strength, and stiffness in the absence of colVI [101]. Lack of adequate muscle stimulation in patients with BM and UCMD could play a role in the abnormalities found in tendons, both structurally and functionally [101].

Of note, the role of colVI in tendons was also confirmed in the *Col6a3^hm/hm^* mouse model, designed by Pan and colleagues [102] in order to ablate specifically the production of the α3(VI) and resulting in the almost complete absence of colVI tetramers secretion in tissues and by fibroblasts in vitro [102]. Similar to what was observed in *Col6a1^−/−^* mice, colVI absence determined an alteration in the assembly of collagen I fibrils that appeared highly dishomogeneous in terms of cross-sectional diameters, with a prevalence of smaller fibers, compared with wild-type tendons. This was particularly evident in the pericellular region of tendon fibroblasts, supporting the idea of a role for colVI in orchestrating the assembly of collagen fibrils within fibroblasts’ pericellular micro-domains [102]. Whether these alterations are directly mediated by the absence of colVI in the extracellular space or by the modulation of other ECM proteins found to be altered in their expression in colVI null tenocytes [101,102] remains to be defined.

### 6.3. Clinical Studies

Patients with collagen VI-related myopathies have axial and proximal joint contractures, as well as distal joint hypermobility, indicating that tendon function is involved in addition to muscle weakness. Therefore, in addition to intensive physiotherapy aimed at stretching and recovery of joint ROM, orthopedic surgery plays a key role in recovering mobility and improving quality of life, with surgeries such as Achilles tendon lengthening. Available clinical studies are anecdotical but give a clear insight of the pathology of these diseases [10].

Antoniel et al. [98] examined biopsies of the pedidium tendon from a UCMD patient with a heterozygous *COL6A1* mutation and of the piriformis tendon from a BM patient with heterozygous *COL6A2* mutation who had a femur fracture surgeryand compared them with two biopsies of similar tendons from controls who had surgery for other reasons. In the control cultures, the colVI network appeared well arranged and attached along the cell surface, thus ensuring cell attachment to the substrate. The UCMD and BM cultures showed a disordered colVI network and limited interaction with cellular processes [98]. The controls exhibited an even distribution of colVI filaments along tendon fibroblast processes, whereas in the BM and UCMD cultures, colVI exhibited mostly intercellular distribution. In comparison to the filamentous arrangement of the controls in long-term patient cultures, colVI organization appeared spotty [98]. ColVI organization was altered in tendon cultures from both BM and UCMD patients using immunofluorescence. In fact, colVI formed evident clumps in the ECM of patients’ cells and did not appear to be connected with the cell surface. The immunofluorescence pattern was validated by rotary shadowing analysis, which revealed that colVI aggregates were made up of tangled microfilaments that were hardly linked to the cell processes [98].

According to previous studies on muscle and cutaneous cultures, changes in colVI expression differ between BM and UCMD. In BM, there are changes in protein organization, detected by rotary shading and electron microscopy, while in UCMD, there is an absence or reduced production of colVI. Consequently, the severity of colVI abnormalities observed in BM tendon fibroblast cultures could indicate that colVI mutations affect protein organization differently depending on the tissue [98].

In another study [103], peroneal tendon biopsies were taken from two healthy patients (17 and 21 year of age) during foot surgery, and from a previously genetically described UCMD patient during a talar-calcaneal extra-articular fusion (Grice procedure). Ultrastructurally, the tendons of the controls had tenocytes with long cellular processes and aligned and well-packed collagen fibers. The PCM of tenocytes contained collagen fibrils that were tightly connected to the cell surface. In the tendons of the patients, on the other hand, tenocytes showed reduced cellular processes and typical features of necrotic cells, with an abnormal accumulation of microfibrillar/reticular material in the PCM. Cross-sectioning of the collagen fibrils showed a smaller mean fibrillar diameter than in the controls. The effect of colVI deficiency on tendon matrix organization in the normal and UCMD patient tendon cultures was investigated using immunofluorescence microscopy. In the UCMD tenocyte culture, colVI was significantly reduced and weakly linked to the cell surface. In tendon sections and tenocyte culture from the UCMD patients, colVI was extremely low, in agreement with the low levels and interrupted network of colVI observed at the skeletal muscle level [103].

The BM/UCMD tenocyte cultures not only were important to describe in vitro alterations and ultrastructural differences in collagen deposition and ECM organization but also to unravel more mechanistic insight related to how colVI impact in this cell type is mediated intracellularly. Indeed, a study conducted in tenocyte cultures from healthy patients’ biopsies revealed that CSPG4/NG2, a major transmembrane proteoglycan, is the receptor able to mediate cell contact to the secreted colVI and to modulate cell migration [104]. The same colVI–NG2 axis appeared disrupted in tenocytes derived from the BM and UCMD patients bearing mutations in different colVI genes, showing impaired migration upon in vitro scratch wound assays, thereby highlighting a further relevance in injury response [98].

ColVI deficiency negatively affects tendon matrix organization both in vivo and in vitro, similar to the changes observed in Ehlers–Danlos syndrome, suggesting a similar pathophysiological mechanism [105,106].

Reduced load, disuse, and sarcopenia due to aging were believed to cause the fibrillar abnormalities. The presence of alterations in the ECM of patient cultures reduced the importance of muscle dysfunction as a determinant of the tendon phenotype [104,107,108].

## 7. Cartilage

### 7.1. General Aspects

Cartilage surrounds the articular surfaces and reduces friction, allowing for painless movement of synovial joints. It is made up of chondrocytes that are integrated in an ECM that is made up of a macromolecular framework and water. Its response to trauma is very limited due to its avascular, aneural, and hypocellular structure. Chondrocytes are primarily responsible for articular cartilage homeostasis. Collagen, thanks in part to its interaction with proteoglycans and interstitial fluid components, ensures the mechanical properties of cartilage [109,110].

ColVI is a prominent component of the PCM compartment in articular cartilage, which is important in the physiology of the synovial joint and has an indirect role in the mechanical environment of chondrocytes [89]. ColVI deficiency can lead to osteoarthritis progression due to either loss of PCM characteristics.

Actually, the amount of colVI in the articular cartilage is estimated to be only 1% of the total collagen species [111]. Here, it is mostly found in the PCM of adult cartilage, where it helps chondrocyte adhesion and integrity [3]. PCM completely surrounds articular cartilage cells and governs the biomechanical, biophysical, and biochemical signals sensed by the chondrocyte. PCM plays an important role in either protecting cells or acting as a filter or transducer of physical signals in the ECM, possibly through an interaction of colVI with integrins or other cell surface receptors. Many ECM components and the cell membrane have a high affinity for colVI, as it is essential in mediating cell–matrix and intermolecular interactions in a variety of tissues and cell cultures. In articular cartilage, colVI interacts with hyaluronan, decorin, and fibronectin to form a network that connects the chondrocyte to the PCM [89]. It is thought to play a role in cell anchoring and matrix cell signaling and is most likely a contact between the hard interterritorial cartilage matrix and the chondrocyte [112]. As a result, colVI serves a dual purpose in chondron integrity preservation: (I) it stabilizes collagens and proteoglycans of the pericellular microenvironment, by connecting to the radial collagen network; (II) it ensures that the PCM and cell nucleus are securely attached, thanks to specific cell surface receptors mediating its interaction with chondrocytes [111].

Interestingly, increased colVI was observed in osteoarthritic cartilage [113], where the PMC derangement is expected to have an impact on chondrocyte metabolic activity [114,115]. The type VI in the milieu could possibly play a role in the creation of chondrocyte clusters, as the PMC is thought to be a key factor in chondrocyte proliferation, which is one of the hallmarks of osteoarthritic cartilage [112].

### 7.2. Animal Models

Several studies found new evidence of severe articular cartilage alterations in *Col6a1^−/−^* mice [89,116].

A reduction in cartilage stiffness, despite a structurally intact PCM, was observed in the absence of colVI. Furthermore, colVI deficiency results in chondrocyte swelling and affects osmotic signal transmission, thus highlighting the role that collagen VI plays in the transmission of mechanical and osmotic stresses from the ECM to the PCM and then to the chondrocytes [116].

Alexopoulos et al. [89] used *Col6a1^−/−^* animals to see if a lack of colVI affects PCM formation and biomechanical function, as well as the mechanical environment of chondrocytes during hip joint loading. In this study [89], mice lacking colVI showed no signs of disease and had similar survival to wild-type mice. The articular cartilage of wild-types had pericellular staining for colVI, which was absent in knockout mice. In knockout mice, the semi-quantitative histologic study of cartilage deterioration indicated significant age-dependent osteoarthritic changes. *Col6a1^−/−^* mice exhibited cartilage with linearly elastic PCM and a significantly lower Young’s modulus of chondrons than wild-type [89]. As the *Col6a1^−/^*^−^ mice aged, they developed osteoarthritis more quickly [89]. These findings imply that early changes in the mechanical properties of the PCM may be related to the progression of osteoarthritis [89].

Another study looked at colVI role in the morphology and physical characteristics of cartilage in the knee joint of *Col6a1^−/−^* mice. *Col6a1^−/−^* mice showed delayed or reduced cartilage deterioration with age compared with *Col6a1^+/+^* mice, unlike the hip joint, but significantly greater and earlier osteophyte formation. Physical characteristics of articular cartilage (elastic modulus, coefficient of friction, roughness) did not differ consistently between genotypes. Thus, the lack of colVI on synovial joint health and function may depend on the specific site [78].

Not only limb-related articular compartments but also intervertebral disks (IVD) were the object of studies in ovine and murine samples [117]. IVDs are fibrocartilage cushions located between the respective superior and inferior facets of the vertebral articular processes as well as through the joints of the vertebral bodies. The inner nucleus pulposus, the outer annulus fibrosus, and the cartilaginous endplates that anchor the discs to adjacent vertebrae are the three major components of the IVD [118,119]. The first is a gel-like tissue in the center of the IVD, accounting for much of the spine’s strength and flexibility and composed mostly of collagen and proteoglycans. It is surrounded by the annulus fibrosus, containing an inner and an outer portion differing primarily in their collagen composition and morphology of ECM-secreting cells [120]. Anulus fibrosus is organized in 15 to 25 lamellae, interspersed with proteoglycans, glycoproteins, elastic fibers, and connective tissue cells. Lamellae are linked together by translamellar cross-bridges, and the number of these bridges per unit area strikes a compromise between strength and flexibility [121].

By immunofluorescence on intervertebral disc tissue sections, colVI was found in the PCM close to the disc cell borders in the outer and inner anulus fibrosus and nucleus pulposus. ColVI exhibited a diffuse distribution from the cell margin to the edge of the chondrons surrounding the disc. ColVI also joined strings of cells in the outer fibrous annulus situated along the primary axis of the type I collagen fiber bundles laid in the annular lamellae and was also an important component of translamellar cross-bridge networks in ovine and murine IVDs. Translamellar cross-bridge abundance in lumbar ovine IVDs also showed age- and spinal level-dependent trends. In addition, the relative size of the transverse bridges and their numbers decreased significantly with advancing age [117].

Of note, articular alterations were reported also in zebrafish in the absence of colVI [57]. In zebrafish, colVI was found to be highly expressed, starting from 48 h post-fertilization and still detectable at six days post-fertilization at the level of palatoquadrate and ceratohyals cartilages [122]. Of note, development of craniofacial cartilaginous compartments is particularly conserved from fish to humans [123] and in accordance to what was described in the mouse, colVI was found during cartilage morphogenesis to stain PMC of chondrocytes and perichondrium [122]. When colVI is lacking, the arrangement of ceratohyal angles appear to vary in an age-dependent manner, from narrower angles in the embryos to larger in the adult, in comparison to the wild-type condition. While the presence of an alteration is relevant for clinical considerations, such a contrasting phenotype dependent on the developmental stage is in line with the start of eating behavior that in zebrafish occurs approximately seven days post-fertilization, therefore potentially being an effect of the differential force and functioning exerted by muscles and tendons in the colVI knockout context [57].

### 7.3. Clinical Studies

Despite the reduced number of specific research studies on cartilage with a major relevance for BM and UCMD patients, the presence of hyperlaxity in distal joints was one of the first recognized diagnostic features for UCMD patients [68] and, although to a minor extent, present in some BM patients [124,125] as well. This became useful in a differential diagnosis with Emery–Dreifuss muscular dystrophy [126].

By the way several reports were focused on human studies aimed at understanding the role of colVI in osteoarthritis.

Pullig and colleagues [127] studied the expression of colVI in normal and osteoarthritis knee cartilage. They showed an increased expression of colVI in osteoarthritis with a different distribution of colVI among different cartilage zones (superficial, central, and deep) compared with normal cartilage, thus suggesting a potential role of colVI in the pathology development [127].

A similar result was found by Nugent et al. [128]; they showed a more widespread diffusion of colVI in osteoarthritis cartilage. Chondrocytes showed upregulation of colVI as a response to altered endoplasmic reticulum function present in the osteoarthritis [128].

Recent approaches for cartilage repair and regeneration have examined the abilities of soluble colVI to stimulate the proliferation of chondrocyte cultures [129]. This may open the way toward using soluble colVI as a useful biologic booster to the proliferation of scarce sources of chondrocytes and subsequent autologous implantation (ACI) [129].

## 8. Ligament

### 8.1. General Aspects

Ligaments are strong structures composed of dense fibrous connective tissue that connect two bones or two parts of the same bone together with a stabilizing function [1].

Ligaments share many properties with tendons, and little is known about the molecular differences between them. Recent studies have focused attention on the developmental and differentiation at the bases of ligaments and tendons [130].

As previously described in tendons, Sardone et al. showed that colVI is associated both in vivo and in vitro with the cell membrane of ligament fibroblasts, extending along with their processes and forming a fibrillar PCM [131].

With relevance for colVI in ligaments, the *posterior longitudinal ligament* and the *ligamentum flavum* were the specific object of some studies on their pathological deterioration involving ECM alteration [132,133]. The first one runs along the posterior aspect of the vertebral bodies inside the vertebral canal, from the body of the axis to the sacrum [134]. The ligament is composed of longitudinal fibers: the denser fibers are deeper and span one vertebra while the superficial fibers span three to four vertebrae [135]. Fibers are wider at the intervertebral spaces and are more adherent to the annulus fibrosus of the intervertebral discs than to the vertebral body. The *ligamentum flavum* is a two-layered structure that bridges the interlaminar gap between consecutive segments. It begins on the ventral side of the suprajacent lamina and inserts on the superior edge of the subjacent lamina. The ligamentum flavum owes its name to the particular yellowish appearance due to the high concentration of elastin and other collagen fibers, including colVI.

In contrast with the relatively rich literature regarding the composition and the biomechanical proprieties of the intervertebral disc, the *posterior longitudinal ligament* and the *ligamentum flavum* need further investigations to characterize the presence and the role of colVI.

### 8.2. Animal Models

There are few studies on this subject in the literature, indicating a topic that still has potential for new research. It may be thought that colVI plays a similar role in tendons and ligaments due to their similar composition [130].

### 8.3. Clinical Studies

Sardone et al. [131] studied the anterior cruciate ligament (ACL) and observed that the distribution of colVI at the cell surface depended on the expression of NG2 proteoglycan, a receptor of colVI. They also found how treatment of ACL fibroblast cultures with an anti-NG2 antibody altered the colVI distribution; thus, NG2 proteoglycan mediated the organization of colVI in the PMC. In vitro, mechanical stresses reduced NG2 proteoglycan levels, altering the binding between colVI and the cell surface and modified the cell cycle. They suggested that ligament injury altered the cell–ECM interaction, leading to poor fibroblast self-renewal capabilities and, thus, poor ligament regeneration [131].

An interesting study of Kawahara and colleagues focused on an increase in colVI in the pathological ligamentum flavum. They demonstrated by morphological and quantitative methods that colVI is a major component of cicatricial and hyalinization thickening of the ligamenta flava following rupture of the normal elastic fiber framework [132].

In controls, colVI was detected immunohistochemically in the collagen among elastic fibers and was weakly colored. Elastic fibers were swollen and degenerated near the hypertrophic edge and the collagenous matrix was expanded and hyalinized. ColVI was abundantly and densely marked in hyalinized tissue, mostly among loosely packed thin collagen fibers and around granular elastic fibrils.

Disruption of elastic fibers and hyalinization appears to be linked to colVI in thickened fibrous ligamenta flava [132].

Another interesting study was conducted to clarify the role of colVI in the ossification of the posterior longitudinal ligament of the spine (OPLL) and the ossification of the ligamentum flavum (OLF) [136]. A variety of possible risk factors have been demonstrated to be implicated in OPLL and OLF by epidemiologic studies, including sex, trauma, hormonal imbalance, dietary habits, and diabetes [133,137,138]; however, the genetic background is considered to be a predominant factor in the etiology of these diseases [139,140,141]. Tanaka et al. recently showed that the locus of susceptibility to OPLL was accurately directed to *COL6A1* by a genome-wide linkage and by linkage disequilibrium studies, and seven single nucleotide polymorphisms (SNPs) of *COL6A1* were associated with OPLL [142]. Among these seven SNPs, the intron32 [143] was not only the most significantly associated with OPLL but also associated with diffuse idiopathic skeletal hyperostosis in Japan [142,144].

## 9. Conclusions

The current review aimed at filling the knowledge gap between scientists and clinicians through a transdisciplinary approach to the role of colVI in the musculoskeletal system, giving insights to its tissue-specific functions; current evidence does not show clear guidelines on the management of patients affected by collagen VI-related myopathies.

However, a better understanding of the role of colVI and its interactions to cells in the musculoskeletal tissues could be the key to correct patient-centered management and to pose the basis to a new molecular approach to therapeutics. UCMD, BM, and MM represent a challenging condition for both the patient and the clinicians. Therefore, it is important to create a multidisciplinary group of professionals capable of focusing on the patient’s needs and, above all, be able to communicate in a more effective fashion.

More recent animal models, including zebrafish, could help researchers to improve the relatively poor literature on bone, ligament, and cartilage tissues available so far. Furthermore, in the near future, we trust that this model could be applied at a preclinical stage to discover new therapeutic targets among the downstream of the genetic defects, allowing tests for new drugs. Physiopathology shows that at an early stage, clinical management should focus on intensive physiotherapy aimed at relieving joint contractures through muscle trophism strengthening exercises and tendon stretching. Orthopedic surgeons may contribute by correcting the severe deformities of the spine and extremities, typical of these patients, and through the performance of less invasive approaches to promote a rapid recovery and more effective post-surgical care.

The present study summarized the updated research progress in the area and gave an overview to support further studies and identify potential novel therapeutic strategies and targets in the management of patients affected by collagen VI-related myopathies.

## Data Availability

No new data were created or analyzed in this study. Data sharing is not applicable to this article.
